# Thermo-Mechanical Degradation Behavior of the Base–Subgrade Interface in Airport Pavements: A Sequentially Coupled Cohesive-Zone Study

**DOI:** 10.3390/ma19122541

**Published:** 2026-06-12

**Authors:** Weihong Yan, Chengchao Guo, Xinrui Li, Wenqiang Zhang, Yiteng Wang, Lei Qin, Leiyang Pei

**Affiliations:** 1 School of Water Conservancy and Transportation, Zhengzhou University, Zhengzhou 450001, China; 2 Henan Province Airport Group Co., Ltd., Zhengzhou 476005, China; 3 School of Civil Engineering, Sun Yat-sen University, Zhuhai 519082, Chinaqinlei5@mail.sysu.edu.cn (L.Q.); peily6@mail2.sysu.edu.cn (L.P.); 4 School of Infrastructure Engineering, Dalian University of Technology, Dalian 116024, China; 5 Department of Architecture, Built Environment, and Construction Engineering, Politecnico di Milano, 20133 Milan, Italy

**Keywords:** airport pavement, thermo-mechanical coupling, cohesive zone model, interface debonding, acoustic emission, digital image correlation

## Abstract

**Highlights:**

**What are the main findings?**

- Thermo-mechanical coupling advances first debonding from 0.04993 h to 0.00254 h.
- Mixed-mode initiation governs first damage, with (t_n_/t_n_^0^)^2^:(t_s_/t_s_^0^)^2^ = 0.38:0.62.
- Thermal pre-weakening shifts the interface closer to the damage threshold before wheel loading.
- Simulated average CSDMG and cumulative AE hits show consistent stage evolution.
- The base–subgrade interface should be treated as a temperature-sensitive weak layer.

**Abstract:**

The thermo-mechanical degradation of the base–subgrade interface in airport pavements was investigated using a three-dimensional sequentially coupled finite element framework in ABAQUS 2023, in which progressive interfacial debonding was described by a bilinear cohesive-zone model through the damage variable CSDMG. The results show that thermal loading markedly accelerates interface degradation when combined with moving wheel loads. Compared with the wheel-loading-only condition, thermo-mechanical coupling advances the first damage initiation from 0.04993 h to 0.00254 h and shortens the severe-degradation stage from 1.000 h to 0.00927 h. This acceleration is attributed to a thermal stress pre-weakening effect, whereby constrained thermal deformation partially consumes the available cohesive resistance and shifts the interface closer to the softening threshold before external loading is applied. A decomposition of the mixed-mode initiation criterion further indicates that the first damage event is governed by synergistic normal–shear interaction, with the normalized contribution ratio (t_n_/t_n_^0^)^2^:(t_s_/t_s_^0^)^2^ = 0.38:0.62, showing that wheel-induced shear is the dominant trigger while tensile opening induced by thermal curling provides substantial preconditioning assistance. In addition, a representative normalized comparison between simulated average CSDMG and cumulative AE hit count demonstrates a consistent stage evolution from distributed deformation to accelerated localization and residual stabilization. These findings indicate that the base–subgrade interface should be treated as a temperature-sensitive weak layer in airport pavement assessment, particularly near joints and other discontinuity-controlled regions.

## 1. Introduction

Airport pavement systems operate under severe service conditions characterized by the simultaneous action of high-contact-pressure aircraft wheel loads and significant environmental fluctuations. In rigid and semi-rigid configurations, non-uniform temperature gradients across the slab thickness induce curling and warping deformations. These thermal distortions generate associated thermal stresses that fundamentally alter the magnitude and spatial distribution of critical pavement responses throughout the diurnal cycle [1,2]. While classical analytical and experimental studies have long recognized the significance of thermally induced slab deformation and its interaction with boundary restraints, recent mechanistic investigations and field measurements have further underscored the impact of transient gradients and built-in curling. These factors have been shown to significantly influence the stress state and long-term performance indicators of jointed concrete pavements [3,4].

In the specific context of airport operations, aircraft loading introduces distinct complexities compared to typical highway traffic. Aircraft gear configurations impose significantly higher contact pressures and generate short-duration, high-impact loading pulses. Furthermore, the load path migrates rapidly across the slab as the landing gear traverses the pavement system, creating complex dynamic stress fields [4,5,6]. Consequently, three-dimensional finite element analysis has been widely adopted to capture complex behaviors such as load transfer, joint interaction, and stress concentrations under moving aircraft loads. These advanced numerical tools are essential for supporting robust airfield pavement design and evaluation [7,8].

At the structural level, thermally induced deformation can “precondition” the pavement system prior to the application of wheel loads. This effect is particularly pronounced near discontinuities, such as joints, where stiffness mismatches and geometric constraints amplify traction gradients. A critical, yet often under-quantified, failure mode in layered pavement systems is interfacial debonding, which compromises shear transfer capacity and accelerates localized damage accumulation. Field observations and mechanistic studies indicate that debonding is promoted by differential deformation between layers, and that curling/warping can generate substantial interfacial opening and shear tractions even in the absence of traffic [9,10,11,12,13,14,15].

To model this progressive degradation, cohesive zone models (CZM) have been extensively employed to represent traction–separation behavior, capturing stiffness softening and fracture energy dissipation [16,17,18,19,20]. Bilinear cohesive laws, in particular, offer a practical balance between numerical robustness and physical interpretability, and have been applied to fracture and interface problems in quasi-brittle and pavement-related materials. Recent developments further extend cohesive formulations to better capture interlayer bonding characteristics and mixed-mode effects relevant to pavement interfaces [20,21]. Despite these advances, the combined thermo-mechanical driving mechanisms governing base–subgrade interfacial degradation in airport pavement configurations remain insufficiently clarified. Existing work often focuses on either thermal curling/warping or wheel-loading response in isolation, and only a limited number of studies explicitly connect transient thermal preconditioning with subsequent moving-load-driven interface damage growth [22,23,24,25,26,27].

Recent companion laboratory studies on the subgrade–base interface have shown that interfacial shear failure under complex service conditions is not governed by a single brittle fracture event, but rather by a progressive transition from ductile sliding to strain localization and brittle debonding. Combined DIC and AE observations further indicate that this transition is accompanied by a shift from distributed deformation and gradual energy dissipation to highly localized softening and rapid energy release. These findings provide a useful physical basis for interpreting interfacial degradation using a cohesive-zone framework, because the bilinear traction–separation law is capable of representing both progressive softening and abrupt post-peak loss of load-carrying capacity. Against this background, the present study does not aim to reproduce laboratory shear tests directly; instead, it extends the interpretation to the structural scale by investigating how transient temperature actions and moving aircraft wheel loads jointly drive the initiation and propagation of base–subgrade debonding in airport pavements.

More specifically, the companion direct-shear program showed that the interface response evolves through elastoplastic growth, slip failure, stress recovery, and residual stabilization, while the AE activity shifts from weak distributed signals to concentrated bursts as failure localizes. DIC observations further identified four representative modes—ductile sliding, progressive localization, brittle fracture, and heterogeneous progressive failure—indicating that the essential feature of interface degradation is the transition from distributed deformation and gradual energy dissipation to localized instability. These experimentally observed features justify the use of a bilinear cohesive representation in the present structural-scale model and provide a clearer physical bridge between specimen-scale failure and the simulated CSDMG evolution.

Against this background, the present study focuses on a representative but insufficiently resolved problem in airport pavement mechanics: the degradation of the base–subgrade interface under coupled aircraft wheel loading and transient temperature actions. A three-dimensional sequentially coupled thermo-mechanical finite element framework is established, in which the transient temperature field is first solved and then introduced into the mechanical analysis, while a bilinear cohesive zone model is employed to simulate progressive interfacial debonding. The novelty of the study lies in four aspects: (i) it explicitly couples transient thermal preconditioning with subsequent moving aircraft wheel loading at the base–subgrade interface; (ii) it interprets damage acceleration through a thermal pre-weakening mechanism rather than describing contour evolution alone; (iii) it links the structural-scale CSDMG localization pattern with companion AE- and DIC-based observations to provide physical interpretation; and (iv) it translates the numerical findings into interface-oriented implications for joint vulnerability, thermal condition assessment, and maintenance prioritization in airport pavements. The results are therefore intended to provide a mechanistic basis for the design, assessment, and maintenance of airport pavements exposed to complex service environments.

## 2. Methods

A three-dimensional finite element model of the airport pavement structure was established to investigate the coupled effects of aircraft wheel loading and transient temperature fields. A sequential coupling strategy was adopted, wherein the temperature field was solved in a heat transfer analysis and subsequently imported into the mechanical analysis as a predefined field.

### 2.1. Model Geometry and Material Properties

**Figure 1:**
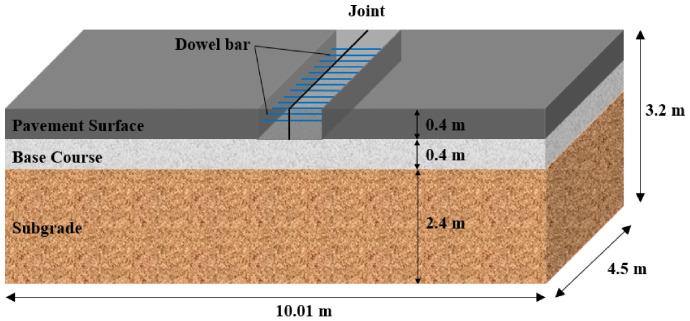
Schematic of the pavement layer configuration and dimensions.

A three-dimensional multilayer finite element model was established in this study, consisting of the pavement surface, base course and the underlying subgrade. The overall dimensions of the model are 4.5 m × 10.01 m × 3.2 m. The pavement system comprises five components: the concrete slab, base course, subgrade soil, dowel bars, and joint sealant. The subgrade is described using an ideal elastic–perfectly plastic Mohr–Coulomb constitutive model, with a friction angle of $30^{\circ }$, a dilation angle of $0^{\circ }$, and a cohesion of 10 kPa. Thirteen dowel bars, modeled using B31 spatial beam elements, are embedded within the concrete slab at a spacing of 0.35 m. All other continuum components are discretized using 8-node linear brick elements with reduced integration (C3D8R) to mitigate shear locking while maintaining computational efficiency. The layer configuration and key geometric parameters are summarized in Table 1 and illustrated in Figure 1.

In the present structural model, the concrete slab and base course were idealized using temperature-independent elastic properties, whereas the subgrade was represented by an ideal elastic–perfectly plastic Mohr–Coulomb constitutive law. This simplification was adopted because the primary objective of the study is to compare the relative debonding evolution under wheel-loading-only and thermo-mechanically coupled conditions within a unified constitutive framework, rather than to establish a fully calibrated rheological model for each pavement layer. In addition, the duration of aircraft wheel passage is short relative to the characteristic time scale of diurnal thermal variation, so the instantaneous structural response to wheel loading can be reasonably approximated at the system level using elastic layer properties.

### 2.2. Bilinear Cohesive Interface Model and Boundary Conditions

**Figure 2:**
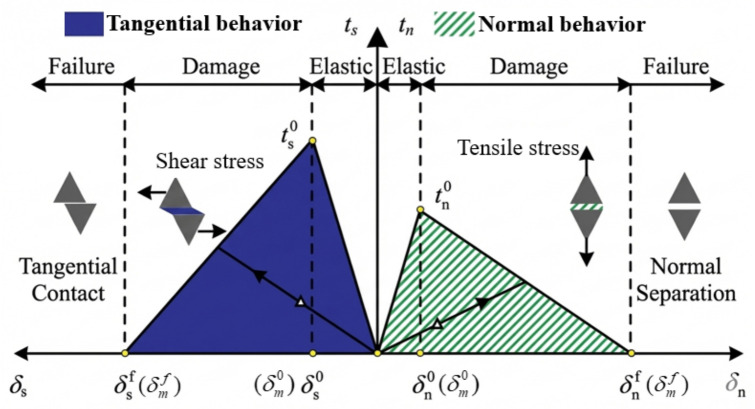
Schematic of the bilinear cohesive-zone interface law adopted in the present study (without additional post-failure frictional contact).

The interface behavior is governed by a bilinear traction–separation constitutive law in Figure 2. In the initial elastic regime, the response is linear, defined by penalty stiffnesses $K_{n}$ and $K_{s}$:

(1) $$t_{n}=K_{n}\delta_{n}$$

(2) $$t_{s}=K_{s}\delta_{s}$$

where, $t_{n}$ and $t_{s}$ are nominal normal and shear tractions, Pa; $\delta_{n}$ is normal separation or opening displacement, m; $\delta_{s}$ is shear sliding displacement along the interface, m; $K_{n}$ is penalty stiffness in the normal direction, Pa/m; $K_{s}$ is penalty stiffness in the shear direction, Pa/m.

When the nominal stress in the tensile direction and the nominal stress in the shear direction of the cohesive element satisfy the following equation, the cohesive element begins to suffer damage:

(3) $${\left(\frac{t_{n}}{t_{n}^{0}}\right)}^{2}+{\left(\frac{t_{s}}{t_{s}^{0}}\right)}^{2}=1$$

where, $t_{n}^{0}$ is normal cohesive strength; $t_{s}^{0}$ is shear cohesive strength.

This condition identifies the onset of interfacial microcracking. When the criterion equals unity, the interface transitions from the elastic regime into the softening regime.

After the cohesive element is damaged, its stiffness degrades, and the degradation pattern is as follows:

(4) $$k_{n}=\left\{\begin{array}{c} (1-D)K_{n},\;\;\;\;\;t_{n0}\geq 0\\ K_{n},\;\;\;\;\;\;\;\;\;\;\;\;\;\;\;\;\;\;\;t_{n0}<0 \end{array}\right.$$

(5) $$k_{s}=(1-D)K_{s}$$

where, $k_{n}$ and $k_{s}$ are the normal and tangential stiffness values of the cohesive element after damage, respectively. *D* is damage factor, which is a function of effective displacement ($\delta_{m}$):

(6) $$D=\frac{\delta_{m}^{f}(\delta_{m}^{\max}-\delta_{m}^{0})}{\delta_{m}^{\max}(\delta_{m}^{f}-\delta_{m}^{0})}$$

(7) $$\delta_{m}=\sqrt{\delta_{n}^{2}+\delta_{s}^{2}}$$

where, $\left(\delta_{m}^{f}\right)$ is the effective displacement at the beginning of the damage; $\delta_{m}^{f}$ is the effective displacement at complete failure; $\delta_{m}^{\max}$ is the maximum effective displacement during the loading process. The cohesive parameters were adopted from published numerical studies with similar interface modeling objectives and were used here to provide a physically reasonable representation of the base–subgrade bonding response [28]. The focus of the present study is not the calibration of a site-specific interface law, but the mechanistic comparison between wheel-loading-only and thermo-mechanically coupled conditions under a consistent parameter set. Specifically, equal penalty stiffnesses were adopted in the normal and tangential directions for the cohesive interface, i.e., $K_{n}=K_{s}=52.20$ GPa/m. The maximum allowable nominal stress in tension was 1.22 kPa, while the maximum allowable nominal stress in shear was 54.97 kPa. The effective displacement was specified as 0.124 mm. In the present ABAQUS 2023 implementation, the interface was modeled as a pure bilinear cohesive interaction, and no additional Coulomb friction law or penalty-based tangential contact was activated after complete cohesive degradation (i.e., when CSDMG approached 1.0). Accordingly, once the cohesive stiffness was fully degraded, the model did not explicitly account for residual post-debonding shear transfer arising from aggregate interlock, surface roughness, or frictional sliding. This assumption was adopted to isolate the cohesive softening-governed degradation process and to ensure a consistent mechanistic comparison between the wheel-loading-only and thermo-mechanically coupled cases. Therefore, the predicted post-failure response should be interpreted as conservative with respect to residual shear resistance, and the late-stage interface slip and damaged-zone coalescence may be somewhat overestimated. To represent realistic support and lateral confinement conditions, boundary constraints were applied as follows: the bottom surface of the subgrade was fixed in all three translational directions (x, y, and z), and normal displacement constraints were imposed on the lateral surfaces of the subgrade, base course, and concrete slab.

### 2.3. Temperature Field Simulation

In this study, a three-dimensional finite element model of the pavement system was developed in ABAQUS with user-defined subroutines. The transient thermal response was simulated by accounting for primary environmental heat transfer mechanisms: solar radiation, surface convection, longwave gray-body radiation, and interlayer thermal resistance. Monthly averaged meteorological inputs, such as air temperature, wind speed, solar irradiation, and sunshine duration, were adopted as the external boundary data to drive the temperature-field evolution of the structure.

**Figure 3:**
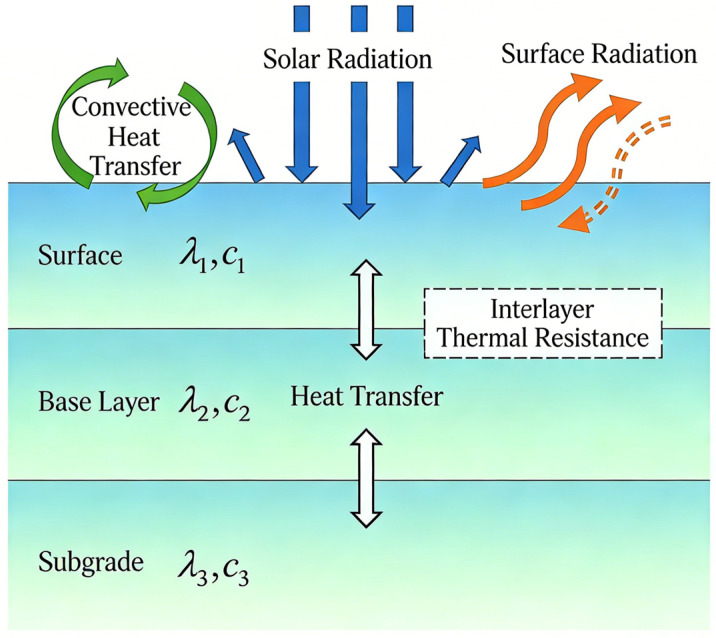
Schematic of temperature field in pavement structure.

Regarding the thermal boundary conditions, the surface thermal loading is characterized by both radiative (second-kind) and convective (third-kind) heat transfer mechanisms. As illustrated in Figure 3, the incident radiative energy comprises: (i) shortwave components, dominated by direct solar radiation and diffuse atmospheric scattering, and (ii) longwave atmospheric radiation. The outgoing radiative energy consists primarily of the pavement’s own gray-body emission. To simplify the calculation, the net longwave radiation is defined as the difference between the pavement emission and the atmospheric back-radiation. Consequently, the upper boundary condition in the ABAQUS model is governed by the superposition of three heat flux components: total shortwave solar radiation, effective net longwave radiation, and convective heat transfer with the ambient air.

(1) Surface convection with ambient air

When air flows over a surface whose temperature differs from the air temperature, convective heat exchange occurs. For the pavement surface, the convective heat flux density can be written as

(8) $$q_{c}(t)=h_{c}\left(T_{s}(t)-T_{a}(t)\right)\times 3600$$

where $T_{s}(t)=T{|}_{z=0}$ is the surface temperature (at z=0), $T_{a}(t)$ is the ambient air temperature, and $h_{c}$ is the convective heat transfer coefficient. The factor 3600 converts $W/m^{2}$ into $J/(h\cdot m^{2})$ when the time unit in the thermal step is hour.

The convection coefficient is correlated with wind speed as

(9) $$h_{c}=3.7w_{v}+9.4$$

where $w_{v}$ is the wind speed (m/s).

To represent the daily cycle and the slight asymmetry of air temperature variation, the air temperature is approximated using a weighted combination of two sinusoidal terms:

(10) $$T_{a}(t)=T_{1}+T_{2}\left[0.96\sin\left(\omega \left(t-t_{0}\right)\right)+0.146\sin\left(2\omega \left(t-t_{0}\right)\right)\right]$$

with $T_{1}$ denoting the daily mean temperature (half the sum of daily maximum and minimum), $T_{2}$ the temperature amplitude (half the daily range), ω=π/12 (rad), t the local solar time (h), and $t_{0}$ the initial phase (set to match the chosen time origin in the simulation).

(2) Total shortwave solar radiation

The diurnal variation in shortwave solar input is commonly close to a half-wave sinusoid on clear days. The total shortwave solar radiation energy per unit area is idealized as a piecewise function:

(11) $$Q_{sr}(t)=\left\{\begin{array}{ll} 0, & 0\leq t<12-\frac{c}{2},\\ Q_{0}\cos\left(m\omega \left(t-12\right)\right), & 12-\frac{c}{2}\leq t<12+\frac{c}{2},\\ 0, & 12+\frac{c}{2}\leq t\leq 24, \end{array}\right.$$

where $Q_{sr}(t)$ is the total shortwave solar radiation energy per unit area (${J/m}^{2}$), $Q_{0}$ is the peak value at noon, c is the actual sunshine duration (h), and m=12/c.

Because the above piecewise expression is not smooth, it may be inconvenient to apply directly as a boundary input for transient heat conduction. Therefore, it can be expressed in a Fourier form to obtain a continuous and differentiable representation:

(12) $$Q_{sr}(t)=\frac{Q_{d}}{m\pi }+\sum_{k=1}^{\infty }a_{k}\cos\left(\frac{k\pi \left(t-12\right)}{12}\right)$$

where $Q_{d}$ is the daily total shortwave solar radiation energy per unit area (${J/m}^{2}$). The coefficients $a_{k}$ are computed as

(13) $$a_{k}=\left\{\begin{array}{ll} \frac{Q_{d}}{\pi }\left[\frac{1}{m+k}\sin\left(\frac{\pi (m+k)}{2m}\right)+\frac{\pi }{2m}\right], & k=m,\\ \frac{Q_{d}}{\pi }\left[\frac{1}{m+k}\sin\left(\frac{\pi (m+k)}{2m}\right)+\frac{1}{m-k}\sin\left(\frac{\pi (m-k)}{2m}\right)\right], & k\neq m. \end{array}\right.$$

In practical computation, the series is truncated at a finite order (e.g., k=30).

If the simulation advances with a time increment Δt (h) and $Q_{sr}(t)$ is assumed constant within each increment, the corresponding shortwave heat flux density can be obtained by

(14) $$q_{sr}(t)=\frac{Q_{sr}(t)}{\Delta t}$$

where $q_{sr}(t)$ is in $J/(h\cdot m^{2})$.

(3) Effective longwave (net) gray-body radiation

Longwave radiation exchange at the surface is modeled using a gray-body approximation. The effective (net) longwave radiative heat flux density between the pavement surface and the atmosphere is written as

(15) $$q_{elr}(t)=C_{0}\varepsilon_{r}\left[{\left(T_{s}(t)+273\right)}^{4}-{\left(T_{a}(t)+273\right)}^{4}\right]$$

where $q_{elr}(t)$ is the effective longwave radiative heat flux density $\left(J/(h\cdot m^{2})\right)$, $C_{0}$ is the Stefan–Boltzmann constant expressed in consistent units, and $\varepsilon_{r}$ is the surface emissivity.

(4) Interlayer thermal resistance

Thermal transfer between adjacent layers is governed by factors such as the thermal conductivity of the interstitial medium, surface roughness, contact pressure, and interface temperature. Since quantifying these effects directly is challenging, interlayer thermal resistance is commonly modeled using an equivalent thermal contact conductance dependent on the interface condition (e.g., gap clearance). To facilitate finite element modeling in ABAQUS, a gap-dependent thermal interaction is defined: (i) under perfect contact (zero gap), heat transfer is governed by the inherent thermal conductivity of the materials, implying negligible interfacial resistance; (ii) when the gap reaches 1 cm, heat transfer ceases, representing infinite thermal resistance (adiabatic condition); and (iii) for gaps between 0 and 1 cm, the thermal conductivity is determined via linear interpolation based on the gap magnitude.

(5) Initial condition treatment

Under periodic diurnal thermal boundary conditions, the internal temperature field converges to a quasi-steady periodic state after a sufficient duration. Consequently, the influence of the initially prescribed temperature field is confined to an early transient stage. Accordingly, the initial temperature is set to a uniform value (e.g., a representative temperature such as 26 °C), and simulation results are extracted only after several daily cycles to ensure the solution is independent of the initial condition. In this study, meteorological data corresponding to the seven hottest days in August in Zhengzhou, Henan Province, China, are selected as the input for the thermal simulation.

### 2.4. Thermal Mechanical Coupling

The aircraft load is modeled based on the dual-wheel main landing gear of a B737-800 aircraft (C-class). Using the principle of equivalent area, the tire–pavement contact patch is simplified as a rectangle, with the load assumed to be uniformly distributed over this area. The simplified load parameters are detailed in Table 2. The loading process is divided into two stages: stress balance and wheel cyclic loading, with a loading duration of 13.604 h. A sequentially coupled approach is utilized, justified by the negligible contribution of mechanical deformation to heat generation in pavement materials (i.e., minimal hysteretic heating). The transient temperature field is pre-calculated and applied as a predefined volumetric thermal load to the structural model. The mechanical analysis comprises two stages: an initial geostatic stress equilibrium step followed by the cyclic moving wheel load simulation. Based on the thermal expansion coefficient of the material, the program will calculate the thermal strain at each integration point:

(16) $$\varepsilon_{th}=\alpha (T-T_{ref})$$

where, α is the coefficient of thermal expansion, T is the current temperature and $T_{ref}$ is the reference temperature (stress free temperature). Due to the limitations of self-weight, interlayer friction, and boundary constraints on the pavement structure, thermal deformation is hindered, resulting in temperature stress. This stress, combined with wheel load stress, constitutes the actual stress state of the pavement.

To further assess the numerical robustness of the damage-softening analysis, the global energy balance was monitored during the mechanical step in ABAQUS. The kinetic energy remained small relative to the internal energy throughout the loading process, with ALLKE/ALLIE below 2.5%, indicating that the response remained effectively quasi-static. In addition, the viscous dissipation introduced by numerical stabilization remained limited, with ALLVD/ALLIE below 0.8%, suggesting that the predicted interfacial damage evolution was not materially affected by artificial numerical damping. Therefore, the simulated initiation and propagation of debonding can be considered numerically stable and physically reliable under the adopted sequential thermo-mechanical framework.

## 3. Result

### 3.1. Interface Response Under Aircraft Wheel Loading Only

#### 3.1.1. Evolution of Interfacial Shear Stress During Wheel-Track Translation

Aircraft wheel loads are characterized by high contact pressure and short-duration, impact-like loading, which leads to a pronounced transient response at the base–subgrade interface as the wheel footprint translates across the joint filler. In the present simulation, a continuous loading duration of 13.604 h is prescribed, during which the wheel load is applied repeatedly for $10^{6}$ cycles; thus, one full loading period corresponds to $1.3604\times 10^{-5}$ h. This extremely high repetition rate produces a periodic but highly localized interfacial stress field that evolves with the moving footprint.

**Figure 4:**
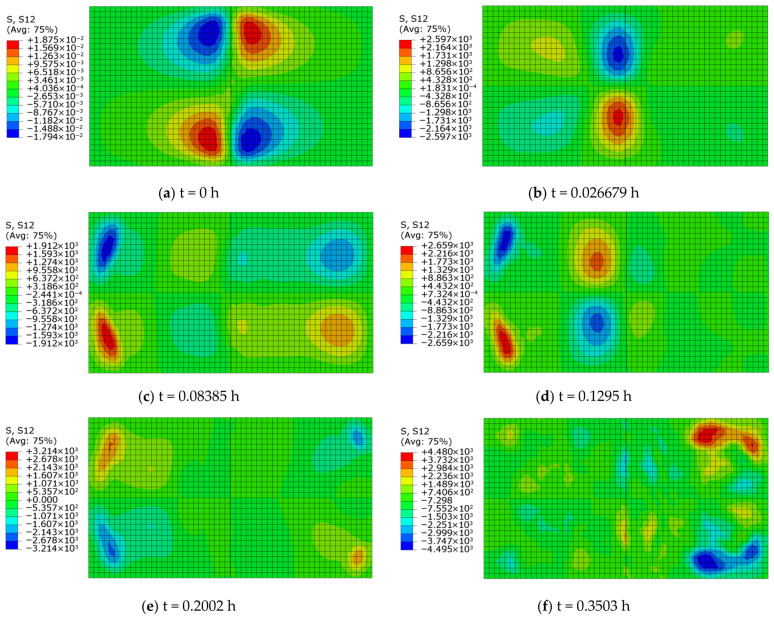
Interfacial shear stress contours at the base–subgrade interface under moving wheel loading at different simulation times.

Figure 4 illustrates the spatial distribution of interfacial shear stress at representative times. As the wheel load approaches and traverses the joint region, the shear stress concentrates near the loaded zone and exhibits an evident “traveling” pattern synchronized with the wheel position. In particular, when the wheel passes the joint filler, the interfacial shear field becomes more heterogeneous, indicating intensified stress transfer and local stress gradients associated with stiffness discontinuities and geometric/boundary effects near the joint. This stress non-uniformity provides a mechanical basis for the subsequent initiation of interfacial damage observed in the cohesive interface elements. Figure 4 illustrates the spatial distribution of interfacial shear stress at representative times. As the wheel load approaches and traverses the joint region, the shear stress concentrates near the loaded zone and exhibits an evident “traveling” pattern synchronized with the wheel position. In particular, when the wheel passes the joint filler, the interfacial shear field becomes more heterogeneous, indicating intensified stress transfer and local stress gradients associated with stiffness discontinuities and geometric/boundary effects near the joint. This stress non-uniformity provides a mechanical basis for the subsequent initiation of interfacial damage observed in the cohesive interface elements.

#### 3.1.2. Interfacial Damage Evolution Under Wheel Loading Only

**Figure 5:**
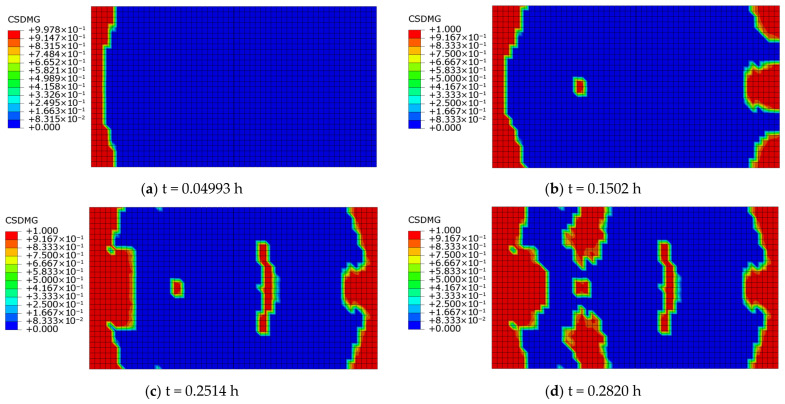

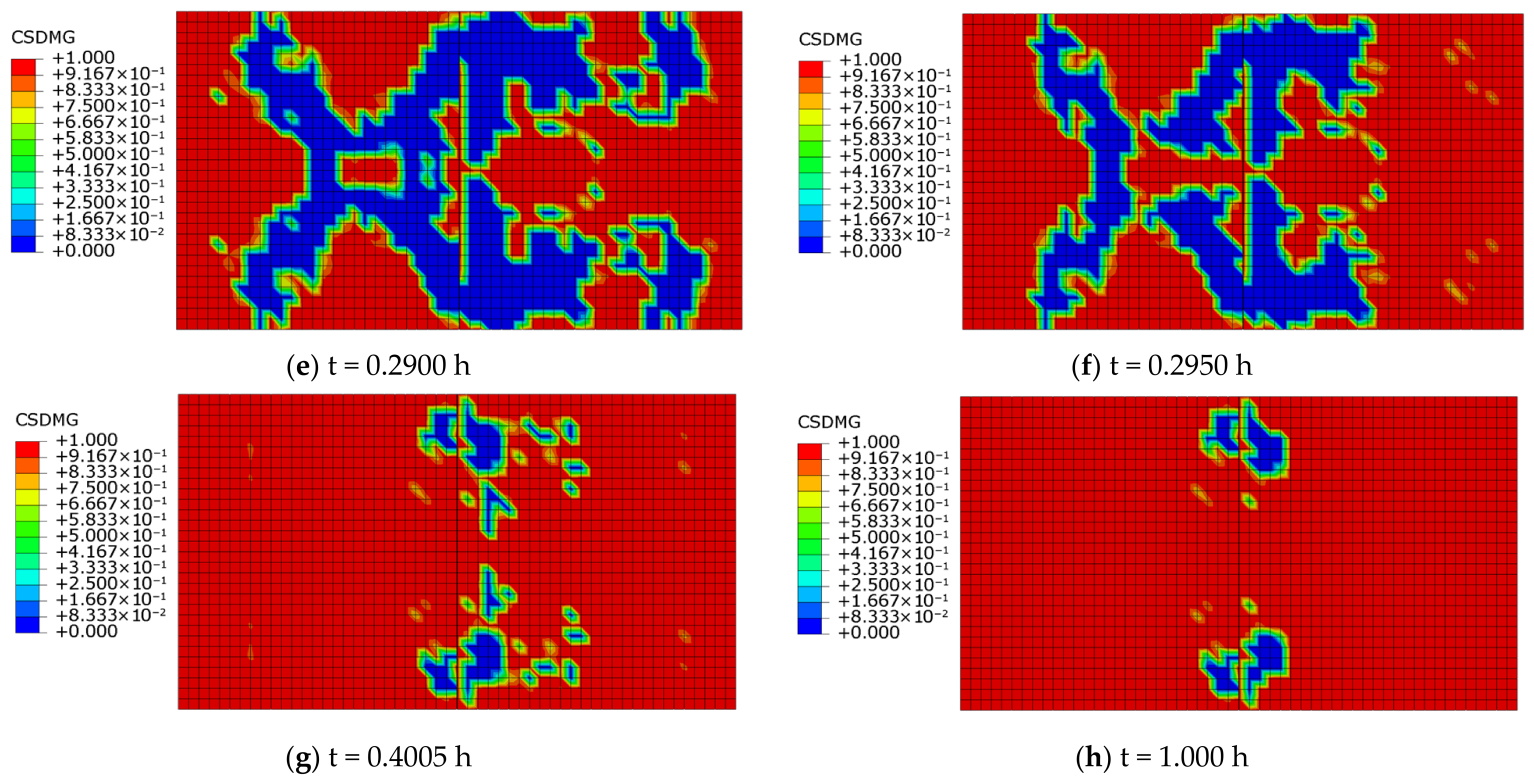
Spatial evolution of the CSDMG at the base–subgrade interface under wheel loading only.

Based on the bilinear traction–separation law within the cohesive zone framework, interfacial degradation is quantified using the scalar damage variable (CSDMG), which represents the overall damage state of the cohesive elements. The results in Figure 5 show a progressive and spatially expanding debonding process under repeated wheel loading.

A key observation is that damage first appears at the interface boundary at t=0.04993 h. This early edge initiation can be attributed to the combined effects of (i) the mechanical mismatch between the base and subgrade layers, and (ii) the repeated tensile–shear perturbations induced by wheel passage. In layered pavement systems, stiffness incompatibility exacerbates localized deformation demands at interfaces; consequently, these regions are highly susceptible to microcrack nucleation and progressive stiffness degradation under cyclic loading. Furthermore, the numerical setup adopts a conservative “worst-case” scenario characterized by uninterrupted wheel loading, which accelerates damage accumulation.

As the loading continues, debonding is also observed on the opposite slab boundary near the joint filler at t=0.1502 h, after which the debonded region expands toward the central interface. By t=0.3 h, a large portion of the interface exhibits debonding, implying that extensive loss of bonding has occurred and that the structure would behave as partially unsupported in the affected zone. Eventually, the interface becomes nearly fully degraded at t=1 h, indicating that the base and subgrade layers have largely lost their bonding constraint and thus show substantially reduced composite action.

It should be noted that, because no additional post-failure frictional contact was introduced after complete cohesive failure, the late-stage reduction in interfacial shear transfer shown in Figure 5 should be interpreted as a conservative estimate. Therefore, the predicted expansion rate of the fully damaged regions may be somewhat faster than that of a rough interface retaining residual frictional resistance.

### 3.2. Interfacial Damage Evolution Under Thermo-Mechanical Coupling

**Figure 6:**
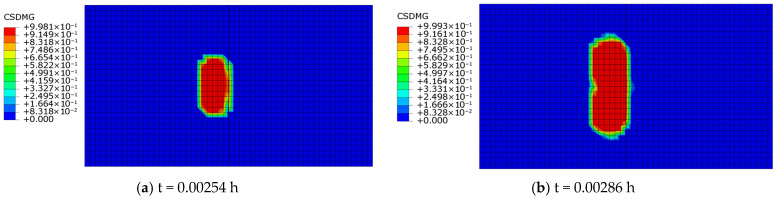

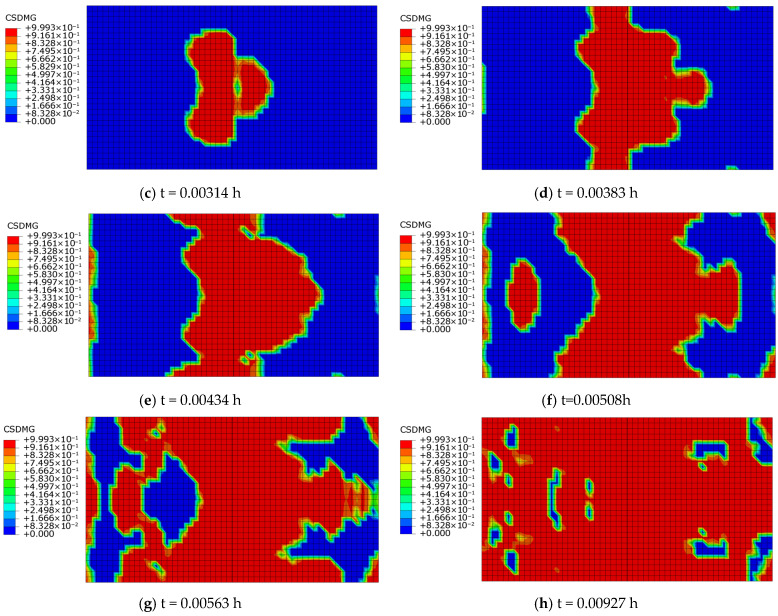
Spatial evolution of the CSDMG under thermo-mechanical coupling.

Figure 6 presents the evolution of the interfacial damage variable when the thermal field is superimposed onto the moving wheel load. Compared to the wheel-loading-only scenario, two distinct coupled effects are evident. First, the onset of damage is significantly advanced. Localized debonding initiates at t=0.00254 h in the central region adjacent to the joint filler. Second, the damaged area expands more rapidly and covers a wider spatial extent for the same nominal time steps. With continued thermo-mechanical action, the central deboned zone propagates across the joint while simultaneously extending toward the interface boundaries. Boundary debonding becomes visible by t=0.00434 h, and by t=0.00563 h, the boundary and central damaged regions coalesce, implying that the interfacial bonding capacity is nearly exhausted.

Mechanistically, this accelerated failure under coupled loading aligns with the well-documented role of temperature gradients in inducing slab curling and warping. These deformations generate significant tensile stresses and additional shear demands at layer interfaces. Thermally induced curvature can cause a partial loss of support or elevate interfacial traction demands even before external traffic loads are applied, thereby increasing the susceptibility to debonding upon wheel passage [29]. Fundamentally, thermal actions do not merely superimpose an additional load component; they alter the stress path and shift the interface state closer to the damage initiation threshold, significantly reducing the number of mechanical cycles required to trigger rapid degradation. This observation corroborates the consensus that curling-related deformation and interface defects (e.g., debonding) interact synergistically in rigid layered systems subjected to environmental gradients [30].

**Figure 7:**
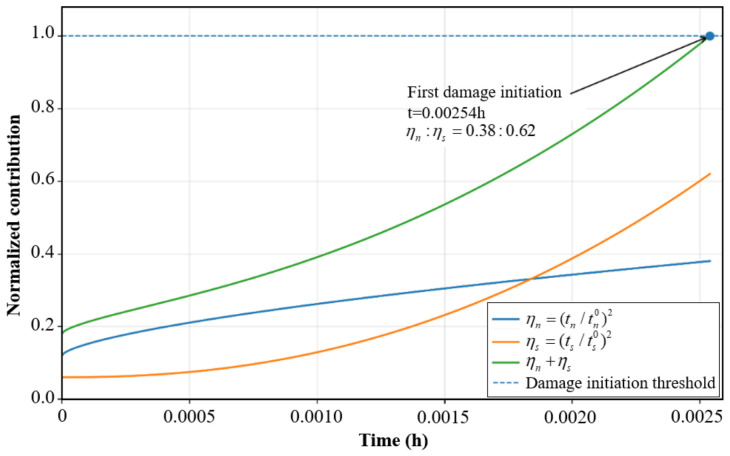
Time evolution of the normalized normal and shear contributions near the joint region at the first damage stage under thermo-mechanical coupling.

To further clarify the physical origin of the first damage event under thermo-mechanical coupling, the quadratic initiation criterion in Equation (3) was decomposed into a normal contribution, $\eta_{n}={\left(t_{n}/t_{n}^{0}\right)}^{2}$, and a shear contribution, $\eta_{s}={\left(t_{s}/t_{s}^{0}\right)}^{2}$, as shown in Figure 7. The results indicate that the normal term increases progressively during the thermal preconditioning stage, reflecting the gradual elevation of tensile opening tendency induced by slab curling/warping near the joint region. By contrast, the shear term remains relatively low at the early stage but rises rapidly as the moving wheel load approaches the critical location. At the instant of first damage initiation (t = 0.00254 h), the interface reaches the threshold condition $\eta_{n}+\eta_{s}\approx 1$, with the relative contribution ratio $\eta_{n}:\eta_{s}=0.38:0.62$. This demonstrates that the onset of debonding is governed by a mixed-mode interaction rather than by pure tensile or pure shear failure alone. More specifically, thermal action primarily acts as a preconditioning factor that raises the normal traction contribution in advance, while wheel loading provides the additional shear increment required to drive the interface to the damage initiation threshold. Therefore, the accelerated debonding observed under thermo-mechanical coupling should be interpreted as the result of synergistic normal–shear interaction, with shear playing the dominant triggering role and tensile opening providing substantial assistance.

### 3.3. Quantitative Comparison of Damage Evolution Under Different Loading Conditions

**Figure 8:**
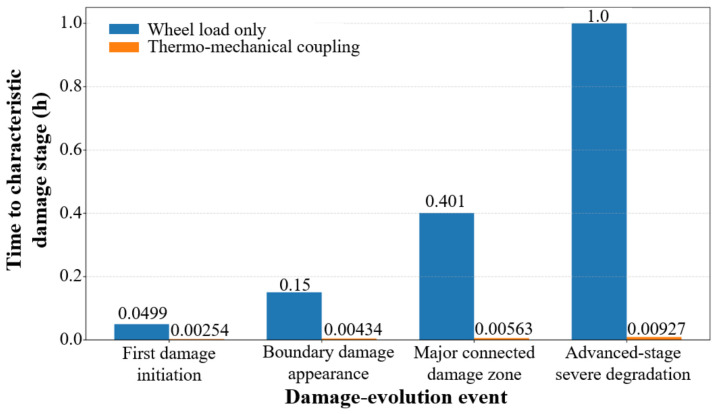
Quantitative comparison of characteristic damage evolution times under different loading conditions.

To further quantify the influence of thermal action on interface degradation, the characteristic damage-evolution times under wheel loading only and thermo-mechanical coupling are compared in Figure 8. Under wheel loading only, the first damage initiation, boundary damage appearance, formation of a major connected damage zone, and advanced-stage severe degradation occur at 0.04993 h, 0.1502 h, 0.4005 h, and 1.000 h, respectively. By contrast, under thermo-mechanical coupling, the corresponding characteristic times are reduced to 0.00254 h, 0.00434 h, 0.00563 h, and 0.00927 h, indicating that the entire debonding process is markedly compressed when thermal loading is superimposed on the moving wheel load.

A direct comparison further shows that thermo-mechanical coupling advances the first damage initiation by approximately 19.7 times, accelerates the occurrence of boundary damage by about 34.6 times, and shortens the formation of a major connected damage zone by roughly 71.1 times. At the late stage, severe interface degradation under coupled loading is reached at 0.00927 h, whereas the wheel-loading-only case requires 1.000 h to approach a comparable damage level, corresponding to an overall time-scale compression of about 107.9 times. These results confirm that thermal action not only accelerates damage initiation, but also markedly intensifies the subsequent propagation and coalescence of interfacial debonding.

These quantitative results confirm that thermal action does not merely introduce an additional disturbance to the interface response. Instead, it fundamentally alters the temporal evolution of debonding by markedly advancing damage initiation and accelerating subsequent propagation. Combined with the spatial observations in Figure 5 and Figure 6, the data indicate that thermo-mechanical coupling changes both the rate and the path of interface degradation, making the debonding process significantly more aggressive than that observed under wheel loading alone.

## 4. Discussion

**Figure 9:**
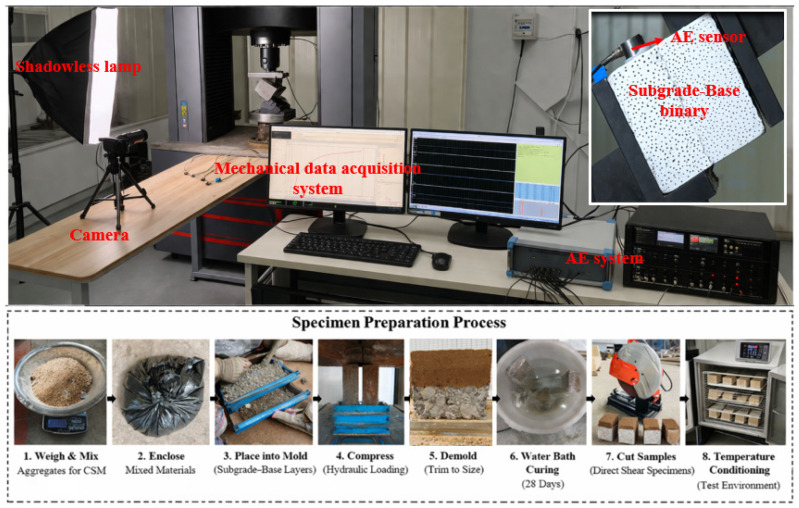
Companion experimental setup of the direct shear–AE–DIC system.

Before discussing the thermo-mechanical degradation mechanism in detail, it should be noted that the following interpretation is not based solely on the present finite element results. It is also informed by companion laboratory shear experiments conducted on the same subgrade–base interface system, in which direct shear tests were combined with acoustic emission (AE) monitoring and digital image correlation (DIC) analysis to investigate the interfacial response under multi-factor coupling conditions. Those experiments revealed that the interface behavior is highly condition-dependent, exhibiting a multi-stage shear stress–displacement response and several representative failure modes, including ductile sliding, progressive localization, brittle fracture, and heterogeneous progressive failure. In the present chapter, these experimental observations are not introduced as an independent research focus, but rather as supporting evidence for interpreting the physical meaning of the simulated CSDMG evolution, thermal stress “pre-weakening,” and localization-controlled debonding under coupled temperature and wheel-loading actions. The companion experimental platform is shown in Figure 9, which provides the physical basis for the AE- and DIC-assisted interpretation cited in the following discussion.

### 4.1. Thermal Stress “Pre-Weakening” of the Interface

A pivotal finding of this study is the thermally induced “pre-weakening” of the base–subgrade interface. In the sequential coupling strategy adopted, the transient temperature field is introduced as a body load. The resulting thermal deformation, constrained by self-weight and boundary conditions, generates significant thermal stresses and interfacial tractions. Within the framework of the bilinear CZM, thermal action need not cause immediate, gross debonding to be detrimental. Even in the absence of wheel loading, thermally induced separation or slip can dissipate a portion of the interfacial fracture energy, thereby reducing the remaining capacity available to resist subsequent mechanical loading. Once the thermally driven relative displacement pushes the traction–separation response into the softening regime, the effective interfacial stiffness begins to degrade. This degradation creates a “self-reinforcing” cycle inherent to softening cohesive laws: damage initiation leads to stiffness reduction, which amplifies relative displacement under constant load, accelerating further damage evolution. Practically, this mechanism implies that the interface may enter a sub-critical, partially degraded state prior to the arrival of the aircraft wheel load. Consequently, when repeated wheel loading is superimposed onto this pre-weakened condition, the interface’ s resistance to shear transfer is compromised, lowering the mechanical threshold for crack nucleation. This interpretation aligns with the simulation results, where interfacial damage initiates earlier and propagates more rapidly in the coupled scenario, as evidenced by the accelerated evolution of the CSDMG.

The decomposition of the quadratic initiation criterion further clarifies the physical origin of the first damage event. At the first damage instant under thermo-mechanical coupling, the normalized contribution ratio (t_n_/t_n_^0^)^2^:(t_s_/t_s_^0^)^2^ = 0.38:0.62 indicates that the onset of debonding is governed by a mixed-mode interaction rather than by pure tensile opening or pure shear sliding alone. The normal component increases progressively during the thermal preconditioning stage, reflecting the gradual elevation of tensile opening tendency induced by slab curling and warping near the joint region, whereas the shear component rises more rapidly as the moving wheel load approaches the critical location. Therefore, thermal action should not be interpreted as causing an independent Mode I failure, but rather as changing the stress path so that a smaller additional shear increment is required for the interface to reach the mixed-mode initiation threshold.

At the same time, several modeling assumptions should be considered when interpreting the results. First, the slab and base course were idealized using temperature-independent elastic properties, whereas the subgrade was represented by an elastic–perfectly plastic Mohr–Coulomb law. Under real diurnal thermal cycles, thermo-viscoelastic relaxation in the pavement layers may reduce the peak thermally induced interfacial tractions, which means that the pre-weakening effect predicted here should be interpreted as a conservative or upper-bound estimate. Second, the interface was modeled using literature-based cohesive parameters rather than material-specific calibrated traction–separation data, so the present framework is more suitable for mechanistic comparison than for direct quantitative prediction. Third, no post-failure frictional contact was introduced after complete cohesive degradation. As a result, the late-stage loss of shear transfer and the coalescence rate of highly damaged regions may be somewhat overestimated relative to a rough interface retaining residual frictional resistance. Finally, the pavement layers were treated as homogeneous and isotropic, and the boundary conditions were simplified; these assumptions may affect the absolute magnitudes and local distributions of stress and damage, especially near discontinuities. Nevertheless, these idealizations do not alter the main conclusion that thermo-mechanical coupling markedly accelerates interface debonding by shifting the interface closer to the mixed-mode damage threshold before wheel loading.

A modeling limitation that should be acknowledged is that the present cohesive formulation does not include an explicit residual friction mechanism after complete bond failure. In real pavement interfaces, aggregate interlock, roughness-induced intermeshing, and frictional sliding can still sustain part of the tangential load transfer after cohesive bonding is lost. From this perspective, the present model is more suitable for capturing the initiation of degradation and the softening-controlled transition toward debonding than for reproducing the fully debonded sliding state in a quantitatively complete manner. In practical terms, introducing a nominal friction coefficient in the range of μ ≈ 0.3–0.6 would be expected to preserve part of the post-debonding shear transfer capacity, delay local slip accumulation, and moderate the coalescence rate of damaged regions, especially in the late stage after CSDMG approaches 1.0. However, the principal conclusion of this study would remain unchanged: thermal action acts as a pre-weakening factor that shifts the interface closer to the damage threshold before wheel loading, thereby markedly accelerating the onset and subsequent development of interfacial degradation.

### 4.2. Stress Superposition and Joint-Driven Localization Under Thermo-Mechanical Coupling

**Figure 10:**
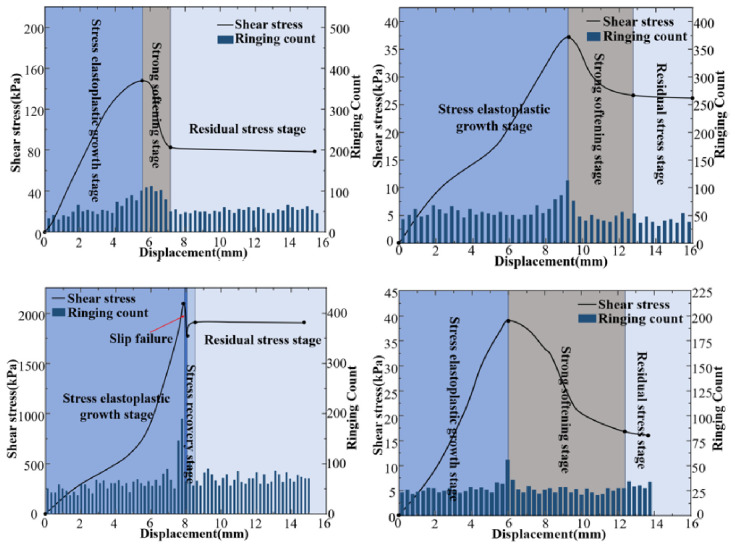
Comparison of representative shear stress–displacement and AE responses under strong and weak interface conditions.

**Figure 11:**
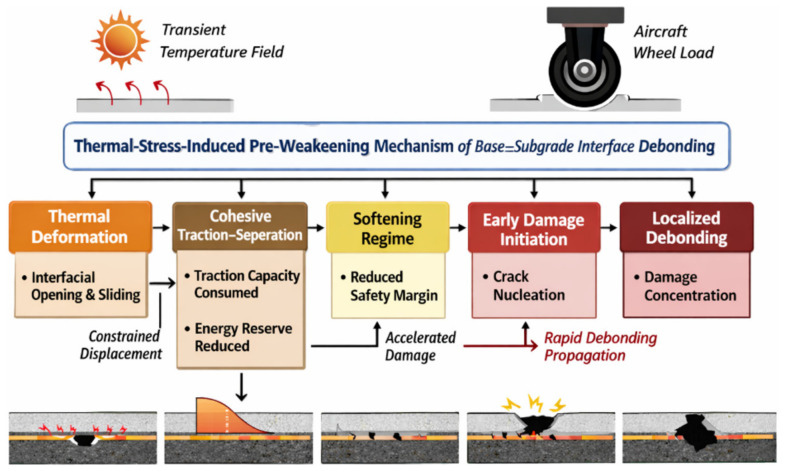
Thermal-Stress-Induced pre-weakening mechanics of base-subgrade interface debonding.

To further illustrate the experimental support for the proposed degradation mechanism, representative shear stress–displacement and AE responses under strong and weak interface conditions are compared in Figure 10. In both cases, the interfacial response shows a multi-stage evolution, but the post-peak characteristics differ markedly. Under strong interface conditions, the specimen accumulates more elastic strain energy before failure and exhibits an abrupt stress drop accompanied by concentrated AE bursts, indicating rapid debonding and localized energy release. Under weak interface conditions, the post-peak stress decreases more gradually and the AE activity is more dispersed, reflecting progressive sliding and distributed energy dissipation. These results show that interfacial failure is governed not only by peak resistance, but also by how the interface enters and evolves through the softening stage. The significance of Figure 11 lies in its support for the thermal stress pre-weakening interpretation proposed in the numerical analysis. Concentrated AE bursts associated with abrupt post-peak stress drop imply rapid localized energy release, whereas dispersed AE activity associated with gradual softening indicates a more distributed dissipation process. This is consistent with the present simulations, in which thermal action shifts the interface closer to the damage threshold so that subsequent wheel loading can drive earlier softening and faster degradation evolution.

**Figure 12:**
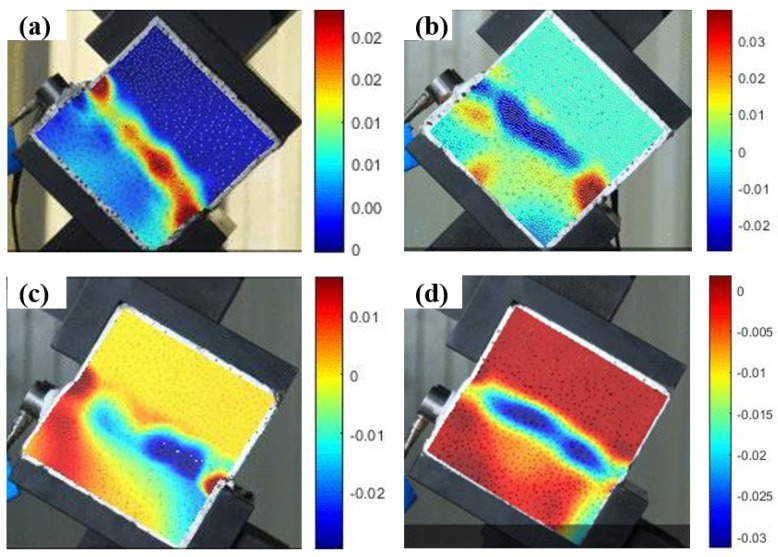
DIC-observed strain localization patterns and representative failure modes of the subgrade–base interface. (**a**) ductile sliding; (**b**) progressive localization; (**c**) heterogeneous progressive failure; (**d**) brittle fracture.

A second key source of experimental support comes from the DIC observations shown in Figure 12. The companion experiments identify four representative failure modes of the subgrade–base interface, namely ductile sliding, progressive localization, brittle fracture, and heterogeneous progressive failure. Although these modes differ in appearance, they share a common trend: deformation evolves from relatively diffuse strain distribution toward increasingly concentrated localization. Ductile sliding is characterized by broad and stable deformation, whereas brittle fracture exhibits a narrow and highly concentrated shear band associated with sudden post-peak instability. The progressive and heterogeneous modes represent intermediate processes in which strain gradually concentrates into a dominant band or multiple local high-strain zones that eventually connect into a non-uniform failure path. In the experiments, the transition from diffuse deformation to concentrated shear band formation reflects the evolution from distributed energy dissipation toward localized instability. In the numerical model, the rapid CSDMG concentration near the joint-adjacent region and the subsequent coalescence of damaged zones under thermo-mechanical coupling reflect the same essential process at the structural scale. Therefore, although the experiments and simulations are conducted at different scales, Figure 12 provides clear mechanistic support for interpreting airport pavement interface debonding as a localization-dominated instability process.

Overall, the companion experimental results corroborate the numerical interpretation that base–subgrade interface degradation is a state-dependent and localization-controlled process. The combined AE, DIC, and shear response evidence indicates that failure is controlled by the transition from distributed deformation and gradual energy dissipation to localized instability and dominant-path formation. This is fully consistent with the present simulations, in which thermal preconditioning reduces the effective resistance reserve of the interface and allows subsequent wheel loading to trigger earlier softening and faster debonding evolution.

**Figure 13:**
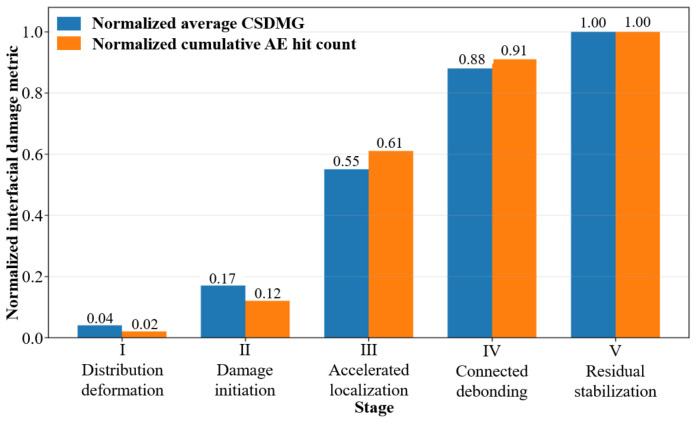
Representative stage-matched comparison between normalized average CSDMG and normalized cumulative AE hit count.

To move the comparison beyond a purely qualitative interpretation, a representative normalized quantitative correlation between the numerical and experimental responses is introduced in Figure 13. On the numerical side, interfacial degradation is characterized by the normalized average CSDMG. On the experimental side, the corresponding failure evolution is quantified by the normalized cumulative AE hit count obtained from the representative companion shear test. Although the finite element model and the shear test differ in scale and loading path, both indicators exhibit the same stage-dependent evolution: an initial distributed deformation stage with slow growth, a damage initiation stage, a rapid localization stage marked by accelerated increase, a connected debonding stage, and a late residual stabilization stage. In particular, the sharp rise in the normalized average CSDMG in Stages III–IV corresponds closely to the rapid accumulation of AE hits, indicating intensified damage localization and energy release. This stage-matched quantitative agreement supports the claim that the CZM framework captures not only the existence of interface degradation, but also the physical transition from distributed deformation to localized debonding.

### 4.3. Engineering Implications for Airport Pavement Design and Assessment

From an engineering perspective, the present results suggest that the base–subgrade interface should be treated as a temperature-sensitive weak layer rather than as an ideal fully bonded boundary. In particular, the marked advancement of damage initiation and the severe time-scale compression observed under thermo-mechanical coupling indicate that analyses based solely on wheel loading may underestimate the onset and propagation rate of interface debonding. This is especially important near joints and other discontinuity-controlled regions, where stress concentration, geometric interruption, and stiffness mismatch make the interface particularly vulnerable.

The findings also imply that airport pavement design and assessment should consider not only nominal peak interface resistance, but also post-peak softening, progressive damage accumulation, and localization-controlled debonding. In practical terms, this means that joints and adjacent boundary regions should be regarded as priority zones for interface strengthening, inspection, and maintenance planning. The results further suggest that thermal condition assessment should be incorporated into structural evaluation, since temperature-induced preconditioning can substantially reduce the additional mechanical demand required to trigger failure. These implications should be understood as mechanism-based design guidance derived from comparative simulations, rather than as direct prescriptive threshold values. Future work should focus on material-specific calibration of cohesive parameters, incorporation of viscoelastic relaxation and residual post-failure friction, and systematic sensitivity analysis of cohesive strengths, interface stiffness, fracture-energy-related parameters, and loading severity, so that the present framework can be extended from mechanistic interpretation toward more quantitative prediction.

## 5. Conclusions

This study established a three-dimensional finite element framework to investigate base–subgrade interface degradation in airport pavements under coupled aircraft wheel loading and transient temperature actions. A sequential thermo-mechanical coupling strategy was employed, utilizing a bilinear cohesive zone formulation to quantify progressive stiffness degradation via the CSDMG. The main conclusions are summarized as follows:

(1) Compared with the wheel-loading-only case, thermo-mechanical coupling advances the first damage initiation from 0.04993 h to 0.00254 h and shortens the severe-degradation stage from 1.000 h to 0.00927 h, demonstrating that thermal action significantly compresses the time scale of debonding evolution.
(2) The decomposition of the quadratic initiation criterion shows that, at the first damage instant, the normalized contribution ratio is (t_n_/t_n_^0^)^2^:(t_s_/t_s_^0^)^2^ = 0.38:0.62. This indicates that the first damage event is not caused by pure tensile or pure shear action alone, but by synergistic normal–shear interaction, with wheel-induced shear as the dominant trigger and thermally induced tensile opening as substantial preconditioning assistance.
(3) The accelerated failure is governed by a thermal stress “pre-weakening” mechanism. By consuming part of the available cohesive resistance through constrained thermal deformation, the temperature field shifts the interface closer to the damage threshold before wheel loading is applied, thereby promoting earlier entry into the softening regime and faster subsequent degradation evolution.
(4) Companion experiments provide consistent mechanistic support for interpreting interface debonding as a localization-controlled instability process. The shear stress–displacement and AE responses indicate that failure is governed not only by peak resistance, but also by post-peak evolution and energy release characteristics, while the DIC observations reveal a transition from diffuse deformation to concentrated strain localization. This is physically consistent with the rapid CSDMG concentration and damaged-zone coalescence obtained in the coupled simulations.
(5) From an engineering perspective, temperature should be regarded primarily as a state-regulating and damage-accelerating factor rather than only a peak-strength-reducing factor. Neglecting environmental preconditioning may therefore lead to non-conservative evaluation of interfacial stability, and thermo-mechanical coupling should be explicitly considered in the design assessment and maintenance planning of airport pavements, especially near joints and other discontinuity-prone regions.
(6) Because the model adopts literature-based cohesive parameters, elastic idealization for the slab and base course, simplified boundary conditions, and no explicit residual post-failure friction, the present simulations are best interpreted as revealing the relative degradation trend and governing mechanisms of interface debonding. Future work should include material-specific parameter calibration, sensitivity analysis, viscoelastic constitutive refinement, and residual friction modeling to improve predictive capability.

## Figures and Tables

**Table 1:** Geometric dimensions and material properties of pavement layers.

Parameter	Pavement Surface	Base Course	Subgrade	Dowel Bar	Jointing Material
Elastic Modulus/GPa	36	1.5	0.08	210	1
Thickness/m	0.4	0.4	2.4	0.6	—
Density/kg·m^−3^	2500	2000	1700	7850	2500
Poisson Ratio	0.15	0.25	0.35	0.3	0.25
Conductivity	8000	6700	6300	1440	360
Expansion Coeff	1 × 10^−5^	9.8 × 10^−6^	4 × 10^−6^	6 × 10^−5^	9 × 10^−5^
Specific Heat	960	910	1040	900	600

**Table 2:** Simplified load parameters of aircraft 737-800.

Main Landing Gear Type	Main Landing Gear Spacing/m	Wheel Spacing/m	Single Wheel Load/kN	Contact Pressure/MPa	Area of Wheel Print/m^2^	Size of Wheel Print	
						Length/m	Width/m
Single axle and two wheels	5.72	0.86	184.54	1.47	0.126	0.427	0.294

## Data Availability

The original contributions presented in this study are included in the article. Further inquiries can be directed to the corresponding author.
